# PD5: A General Purpose Library for Primer Design Software

**DOI:** 10.1371/journal.pone.0080156

**Published:** 2013-11-21

**Authors:** Michael C. Riley, Wayne Aubrey, Michael Young, Amanda Clare

**Affiliations:** 1 Department of Computer Science, Aberystwyth University, Aberystwyth, United Kingdom; 2 Institute of Biological, Environmental and Rural Sciences, Aberystwyth University, Aberystwyth, United Kingdom; University of North Carolina at Charlotte, United States of America

## Abstract

**Background:**

Complex PCR applications for large genome-scale projects require fast, reliable and often highly sophisticated primer design software applications. Presently, such applications use pipelining methods to utilise many third party applications and this involves file parsing, interfacing and data conversion, which is slow and prone to error. A fully integrated suite of software tools for primer design would considerably improve the development time, the processing speed, and the reliability of bespoke primer design software applications.

**Results:**

The PD5 software library is an open-source collection of classes and utilities, providing a complete collection of software building blocks for primer design and analysis. It is written in object-oriented C^++^ with an emphasis on classes suitable for efficient and rapid development of bespoke primer design programs. The modular design of the software library simplifies the development of specific applications and also integration with existing third party software where necessary. We demonstrate several applications created using this software library that have already proved to be effective, but we view the project as a dynamic environment for building primer design software and it is open for future development by the bioinformatics community. Therefore, the PD5 software library is published under the terms of the GNU General Public License, which guarantee access to source-code and allow redistribution and modification.

**Conclusions:**

The PD5 software library is downloadable from Google Code and the accompanying Wiki includes instructions and examples: http://code.google.com/p/primer-design

## Introduction

As experimental DNA sequence manipulations become increasingly complex and large scale genome engineering projects are planned, software is needed for the design and *in silico* evaluation of oligonucleotide primers that satisfy complex criteria. Software packages currently available are most suited for the design of primer pairs for individual PCR applications. Experimental procedures involving multiple sequential PCR steps often require more complex analysis, which is time consuming and error prone using the currently available programs. An automated procedure is the best approach for the complex analyses required. However, a considerable investment of time and effort is needed to build and test the requisite algorithms. The development time can be reduced considerably by a modular method using reusable primer design components from a tried and tested software library, which can easily be employed in the final primer design system.

The most popular freely available primer design software is Primer3 [1], [2]. It consists of open source C code with two web front ends (primer3web and primer3plus). Its purpose is to find suitable primers to amplify a sub-sequence, subject to a range of user defined conditions. Input and output to Primer3 are achieved using text files in a specific file format, which, along with its availability, has made it a popular choice for inclusion in many primer design pipelines as the central primer design tool. In fact, the majority of primer design software now uses Primer3 as a sub-component. Examples include RExPrimer [3], QuantPrime [4], JCVI Primer Designer [5], UniPrime2 [6], BatchPrimer3 [7], PrimerZ [8], ConservedPrimers 2.0 [9] and MPPrimer [10]. Primer3 is flexible with regard to parameters, but limited to simple applications and single primer pairs. More complex applications must be handled by additional software that calls Primer3 as and when necessary.

Those primer design applications that do not use Primer3 usually entail the exploration of more complex issues associated with particular problems. They are packaged as specific applications that solve these particular problems rather than as a general purpose software library and were never intended to have flexible/reusable code. Two examples are Osprey [11] for the design of oligonucleotides for DNA sequencing and microarrays, and Primique [12] for the design of specific primers to distinguish each sequence within a family of DNA sequences. For these applications algorithms were developed to explore issues such as details of thermodynamics and non-specific binding, rather than using the algorithms of Primer3, while at the same time producing families of primers that form a consistent set.

Our development of a new approach to primer design was first inspired by the need to design and analyse large chimaeric primers. These chimaeric primers are used in a multi-stage PCR process for the scar free, seamless deletion of genes in *Saccharomyces cerevisiae* [13]. This method requires the design of three sets of primer pairs: one conventional primer pair of 18–23 bases; one pair that can have primers up to 60 bases in length and a third pair of chimaeric primers, which can be up to 100 bases in length. This method is also required to perform the analysis of dimerisation, annealing temperatures and secondary binding characteristics of all of these primers. The use of Primer3 for this purpose would have been unwieldy, requiring the provision of many additional software components, such as an application to handle primers over 36 bases in length and an application to provide a search for potential non-specific binding locations. Primer3 was not designed as a library of reusable software components, or as a codebase that can easily be extended by the bioinformatics community. It is a single program with many options, around which different wrapper programs can be written, to parse the output and perform further processing. However, the core has now been re-packaged to provide libprimer3 [2], to give programmers closer access. The corresponding header file lists 243 functions at the time of writing. It is possible to make direct calls to these functions and the NCBI Primer-BLAST service does exactly this. We wanted to use a library approach to development from the beginning for PD5, with many small reusable components of code, which is more suited to the problem at hand and would provide increased flexibility, increased ease of maintenance, and be more extensible.

We have developed PD5 to provide a general purpose toolkit of reusable software components suitable for an extensive range of primer design applications. Our aim is for PD5 to be a freely available open source toolkit, to which many community-generated modules can be contributed. In the future we hope that bioinformaticians will more easily be able to re-use the acquired knowledge of primer design, accumulated within the PD5 library.

## Results and Discussion

Here we demonstrate the capability of PD5 with three different example applications all built using PD5 software library components:

1. A command-line or web-based application for discovery of a suitable primer pair to amplify a target region.
2. Design of large chimaeric primers to be used in a 3-stage PCR procedure for the precise scar-free deletion of genes in the genome of *S. cerevisiae*.
3. Design of primers to amplify microsatellite regions to use as markers for a mapping population.

### Discovery of primer pairs

In this example application we provide a command-line interface to the PD5 classes/methods to discover suitable primer pairs for amplification of a target region of a sequence, controlled by a variety of parameters. This command-line application is named *pd5_cli* and is available as part of the PD5 package. This serves mainly as a confirmation of the effectiveness of the PD5 library, and also provides an example for others who want to understand how to employ the PD5 library in their primer design applications. Furthermore, *pd5_cli* can be incorporated into a larger process or script to easily generate a large number of primers automatically without constant user intervention. The output is produced as plain text, HTML or CSV as required by the user. We have used this interface to generate 5127 sets of confirmation primers for seamless deletions of the ORFs in the *S. pombe* genome. These are available at http://www.aber.ac.uk/en/cs/research/cb/projects/pcr-primer-design/.

We have also provided a web interface *PD5Web* (http://PD5web.dcs.aber.ac.uk/), which allows the user to input their template sequence and chosen options. The website then uses *pd5_cli* to produce the primers and provides a web-based display of the results.

This command line application can also be used from within the Galaxy bioinformatics platform [14]–[16]. We have provided the necessary configuration file, and the PD5 wiki page describes the simple setup procedure (essentially, put PD5 folder as a subfolder of Galaxy's tools, and add a few lines to your Galaxy tools configuration file).

### Design of large chimaeric primers for coupled PCR

PD5 is presently being used in the design of primer sets for the precise, seamless deletion of ORFs in the *S. cerevisiae* genome. For this work an application written in C/C^++^ programming language has been developed making full use of the PD5 software library.

This method is based on three PCR reactions employing two different primer pairs to amplify regions of the yeast genome and to construct a URA3-based cassette. The position of one reverse primer is constrained to a fixed location on the template and so, to obtain a reasonable selection of candidates, the length of this primer can range from 30 to 60 bases, with an optimum of 40 bases. The forward primer is selected from a region 500–1000 bases upstream with similar length and GC content as the reverse primer.

Suitable primers for amplifying the URA3 marker gene are also required and these are concatenated with other sequences from the template to make long chimaeric primers. These long primers cannot be designed and optimised using any currently available primer analysis tools. However, the PD5 software library provides modules that are ideally suited to perform this task.

Furthermore, this application is required to automatically select optimum primers for several thousand PCRs without user intervention, and also to be processed using a distributed computer system. Again, the PD5 software library contains the necessary components, which are ideally suited for this.

It was this project that first inspired the development of PD5. Originally, we used Primer3 to find a selection of suitable primers, and BLAST, together with some supporting software, to detect secondary binding and secondary products. Primer3 could not analyse the long primers. We had problems with BLAST in that, to obtain the necessary sensitivity, we used shorter word sizes (4 and 5) and higher E values than recommended by NCBI. This dramatically increased processing time and produced many irrelevant hits, which required filtering by a BLAST file output parser, but still did not detect some significant secondary binding sites (the reasons for this are discussed in the Methods section). These problems were not exclusive to Primer3 and BLAST, hence the development of new primer design methods, which are now available in the PD5 software library.

The PD5 library solves several issues that are not addressed by other general purpose primer design software:

- The detection of potential secondary binding and secondary products was improved by using a sequence matching algorithm optimised for DNA sequences. These methods are included in the PD5 *dna_find* class.
- Existing methods for determining annealing temperature tend to be inaccurate when applied to long primers. To compensate for this, PD5 can simply generate primer pairs of similar length and GC content.
- The chimaeric primers used in the second PCR process can be up to 100 bases in length. The analysis of long primers such as these cannot be done with existing software and so the improved dimerisation analysis methods for long primers, included in PD5, proved invaluable.

The resulting code was executed on a Beowulf computer cluster using Condor [17] and produced primers that were used successfully to delete *S. cerevisiae* genes. In Information S1 (Section S1, Figures S1 and S2, Tables S1, S2 and S3) we provide a sample of the many PCR gel images that we have as evidence for the effectiveness of the primers that were designed for this work. The first gel image demonstrates good results from the first PCR stage for 32 distinct pairs of chimaeric primers targeting 32 different ORFs. This image is part of a larger collection detailing results for the seamless deletion technique, and the full set is to be published separately. The second gel image demonstrates good results for 12 pairs of primers designed to confirm the presence of 12 ORFs in a wild type strain of *S. cerevisiae*.

### Microsatellite markers and flanking primers - PD5_ssr

The PD5 software modules can be integrated easily with other code where required to perform a primer design task. In this instance the task was to find microsatellite regions in the *Miscanthus giganteus* unassembled genome sequence data, and then to design flanking primers for each of these regions so that the microsatellites could be used as potential markers.

There are many existing software tools for the task of locating microsatellites (short tandem repeats of 2 to 6 nucleotides), for example, Sputnik (available from http://espressosoftware.com/sputnik/) and Misa [18]. Indeed, there are even software tools that already integrate the process of finding microsatellites and the process of finding their suitable flanking primers, for example BatchPrimer [7] and PrimerPro (available from http://webdocs.cs.ualberta.ca/yifeng/primerpro/). Our use of PD5 for this task serves the purpose of providing another illustrative use of this library. We demonstrate the flexibility of PD5 and show how it can be integrated with existing applications to facilitate the design of primers for analysing individual microsatellites in the *Miscanthus* genome. For this purpose we developed a C/C^++^ application named PD5_ssr, which integrates PD5 with Sputnik. Sputnik is a public domain application for the discovery of microsatellites and it was chosen for its popularity, speed and simplicity.

We first made minor modifications to Sputnik to wrap it as a C^++^ class, so that its previously hardcoded parameters can be easily changed, and so that multiple Sputnik instances can be created with different parameters. Then we begin by creating an instance of Sputnik with strict parameters, to search for high-scoring microsatellites. When a microsatellite region is found, the Sputnik code makes a call to PD5_ssr to start the search for flanking primers. During the search for primers PD5_ssr makes further calls to new instances of Sputnik (with different, more relaxed parameters) in order to make sure that the primers themselves and the flanking regions up to the microsatellite contain no other microsatellites. In this way the two pieces of software are closely intertwined, which is efficient and avoids the reading and writing of intermediate files, but their interaction remains restricted to just two function calls, and so the software remains maintainable and loosely coupled.

The sequence data comprised 21 Gb of short unassembled sequence reads (averaging approx 500 bases) from a Roche 454 next generation sequencer, equivalent to about three times coverage of the *Miscanthus giganteus* genome.

The constraints for the primer design for this application were as follows:

- Primers to be 20–30 bases in length, for economy.
- Product sizes to be between approximately 80 and 300 bases.
- Products should not contain Ns (frequently found in the reads).
- Primers to have the same GC content, with a preferred annealing temperature of 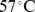 as calculated using the oligoTm code incorporated from Primer3.
- Primers must not contain the microsatellite repeating unit.
- Primers and flanking regions must not contain other microsatellites.
- Primers must not have any non-specific binding within the same read fragment.

In total, 16124 microsatellite regions comprising at least 21 base pairs of repeating units of two, three or four bases, having flanking primers consistent with the above constraints, were produced by PD5_ssr for *Miscanthus*. Their utility is currently being evaluated experimentally. The full code for PD5_ssr is available as part of the PD5 package.

## Conclusion

PD5 is a versatile software library of C^++^ code for developing fast and efficient applications for primer design. As an example of the speed of PD5, the *pd5_cli* command line application can be used to design pairs of confirmation primers for every one of the 5143 ORFs in *S. pombe* in a total of just 29 seconds on a standard desktop Linux machine without a search for alternative binding sites, or 18 hours when we design the primers using a search of the whole genome for potential alternative products to avoid. This software library is freely available as open source under the GPL3 licence. The main advantages in using the PD5 library are the following: -

- The modular design of the software library simplifies integration of PD5 modules with other existing code, and allows further methods/modules to be added as needed.
- The ability to design and analyse long primers (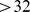 bases).
- The discovery of potential secondary products can be performed automatically.
- PD5 includes specialised methods for the detection of potential dimerisation that out perform existing methods in the analysis of long primers, but remain highly effective with primers of standard length.
- Versatile methods are available for special applications such as the design of primers lacking either cytosine or guanine.
- PD5 provides a non-heuristic method for detection of secondary binding locations.
- PD5 has a versatile prioritised sorting method as well as a multi-objective optimisation approach for the selection of optimum primers. In the event that there are no optimum primers, the PD5 sorting method can select the best available sub-optimal primers rather than none at all.

Although nearly all parameters have default values, an important feature is that all parameters for PD5 can be set by the user. The default values are based on our ongoing work with *S. cerevisiae* and these defaults may not necessarily apply to other organisms. We aim to refine and improve default values and methods from subsequent studies of dimerisation, annealing temperature and secondary binding.

The use of PD5 has already proved to be effective in various challenging primer design applications as exemplified above. Nevertheless, we view the PD5 project as a dynamic environment for the development of primer design software. Therefore, we strongly encourage the bioinformatics community to contribute to PD5: to add to the current collection of modules and to enhance the existing modules. In this way, bioinformaticians will be able to build a repository of knowledge of primer design for reuse by others, in the form of code that has been used in practice in many diverse projects.

The PD5 software library is downloadable from the *primer-design* project pages on Google Code and the accompanying Wiki includes instructions and examples: http://code.google.com/p/primer-design

## Methods

One of the primary objectives of the PD5 project is to produce reusable software modules that provide all the necessary tools for the development of fast and efficient primer design applications. These tools encompass the detection of potential dimerisation, the calculation of annealing temperatures, the detection of potential secondary binding and secondary products, and the optimisation of selected potential primers.

### The PD5 software library

**Figure 1:**
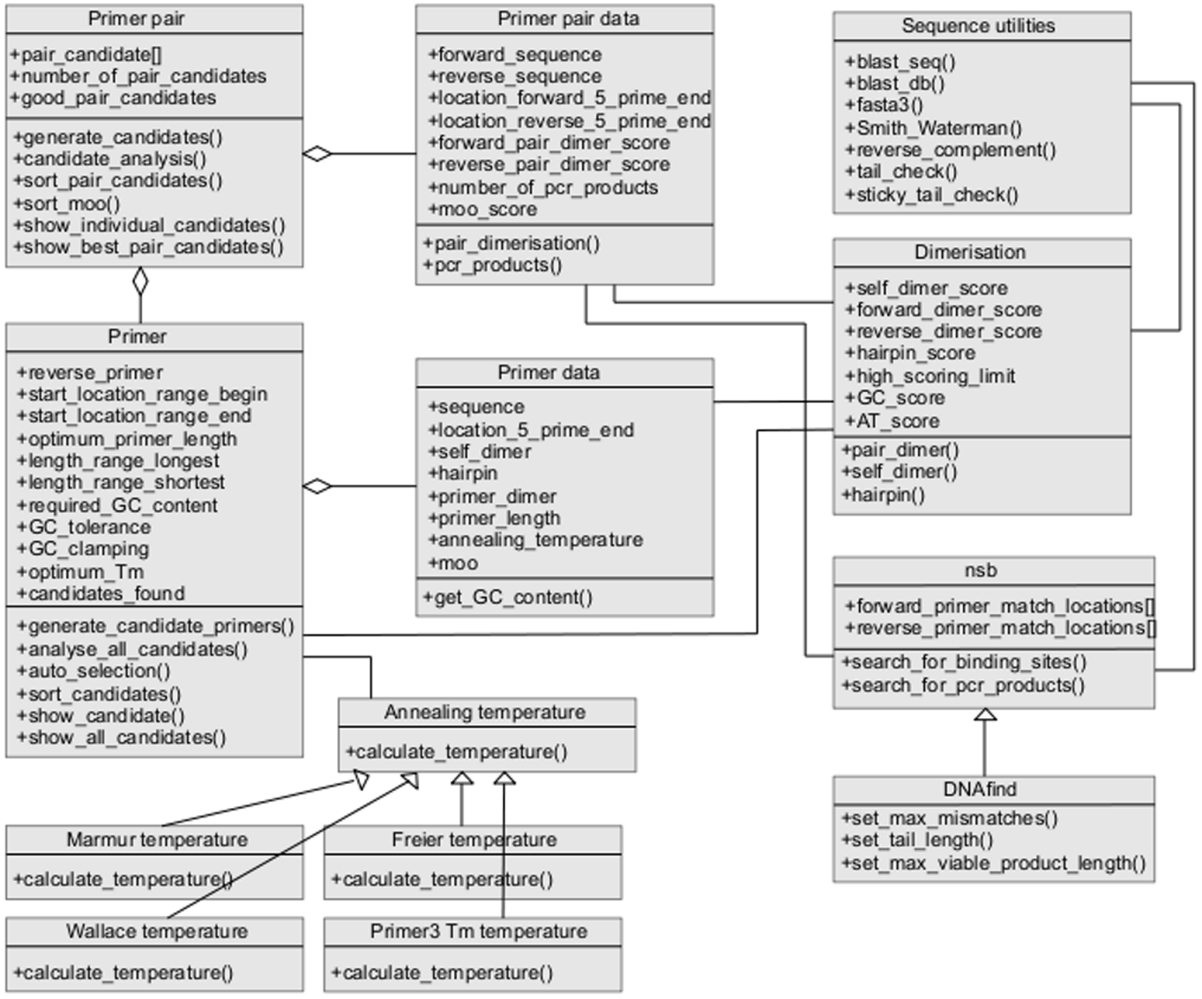
PD5 class structure. The main classes are on the left, the data containment classes in the centre and the analysis classes on the right and below. Within each class only the main public methods and attributes (indicated by + prefix) are shown.

The overall modular class structure of PD5 is shown in Figure 1 where the main classes are on the left, the data classes are in the centre and the analysis classes are on the right. The main classes, *primer* and *primer_pair*, deal with the primer locations, primer candidate generation and primer candidate optimisation for individual primers or for pairs of primers. We expect most users to start with one of these two classes. After setting the various member variables to suit their needs they will call a function to generate candidates, followed by functions to calculate further properties of these candidates and then functions to sort and select the most optimal candidates.

The attributes in the data classes are those required for selection and optimisation of candidate primers. The data classes also contain methods for procuring the data from the relevant analysis class methods. The analysis classes are concerned with evaluating the potential for dimerisation, the numbers of binding sites and products, and the annealing temperatures for all candidate primers.

**Figure 2:**
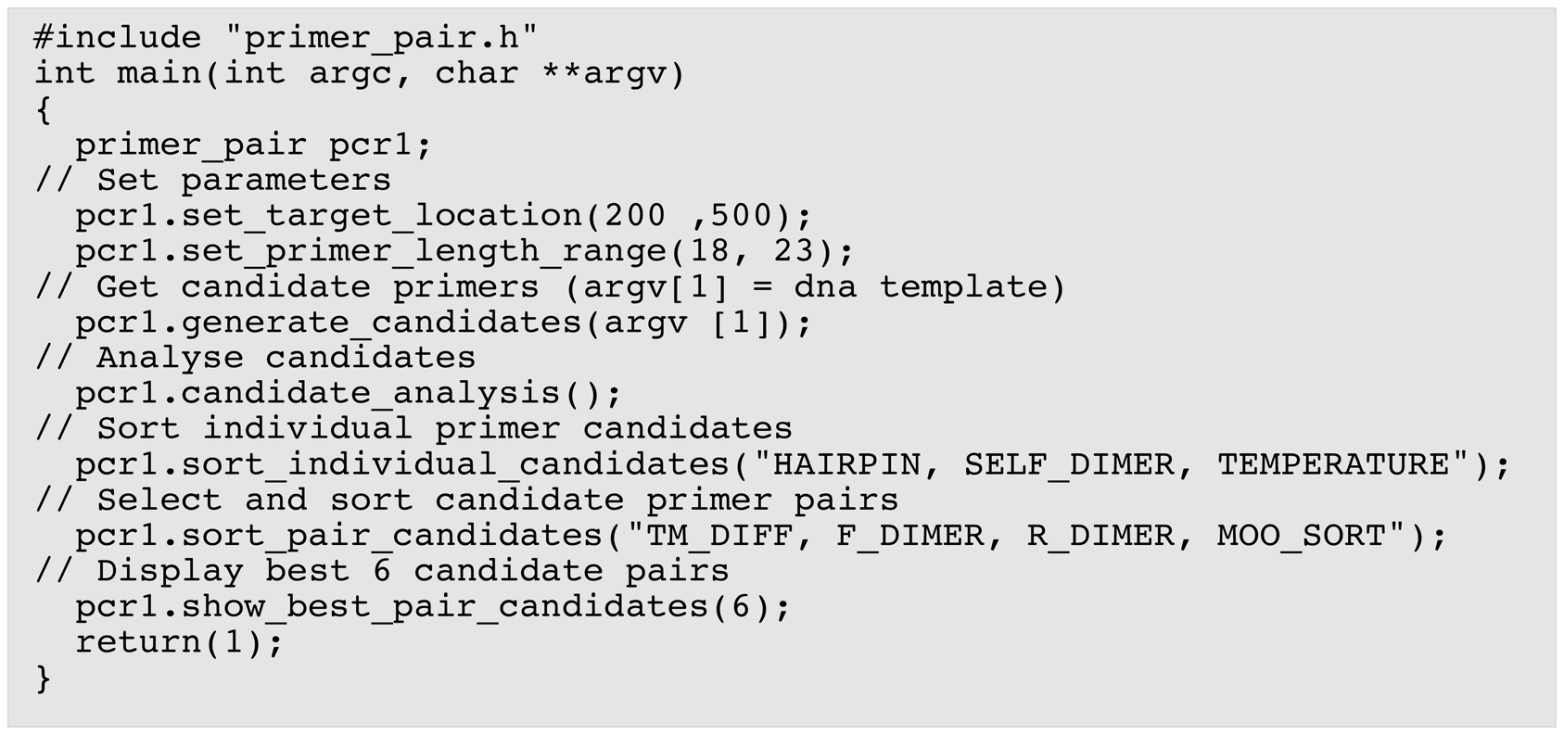
Example code for basic primer analysis using the *primer_pair* class.

**Figure 3:**
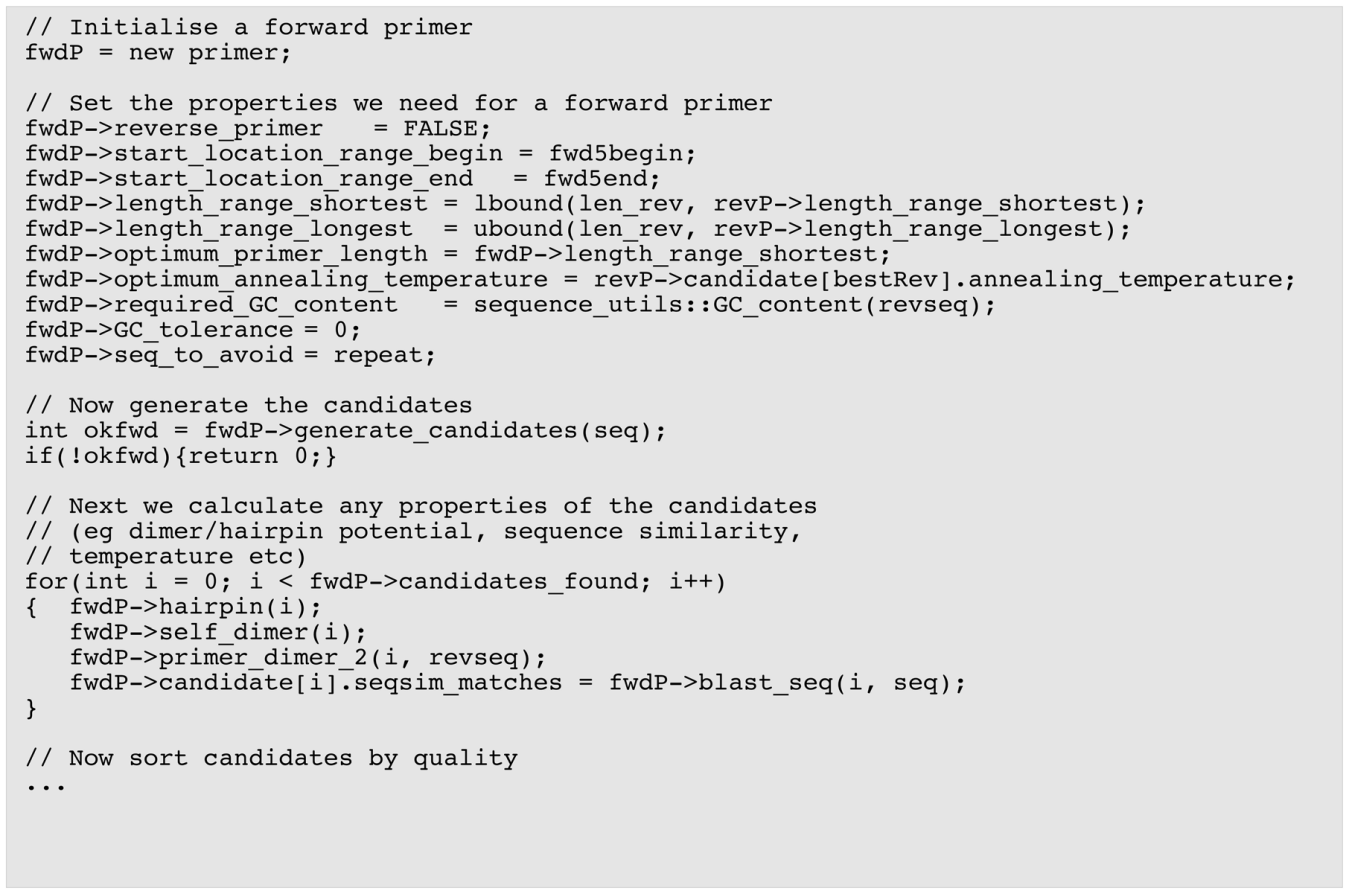
Example code for PD5_ssr, demonstrating some of the selection criteria for the forward primer.

PD5 is designed such that specific classes can be accessed for specific lower level use. For example, the dimerisation class can be used in isolation to check if a particular primer pair might form a pair dimer, or a single instance of *primer_data* class can be used to test annealing temperature, self dimerisation, and hairpin formation for a given single primer. However, the ideal approach for the use of PD5 is that, from a single instance of the *primer_pair* class, the user has all methods available for efficiently designing primers. An example of this is given in Figure 2 showing how simple primer design can be achieved with little more than a handful of function/method calls. An example of more sophisticated primer design is given in Figure 3 showing more detailed design of an individual primer.

### Hairpin formation and dimerisation

Potential dimerisation and hairpin formation are detected using rule based binding affinity methods very similar to those used by Primer3. However, this rule based method is applied to a sequence of a user definable length at the 3′ end of each primer and it is this modification that permits the detection of dimerisation in a much broader range of primer lengths than existing applications. The dimerisation and hairpin methods check the final 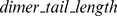 bases at the 3′ tail for complementarity, and give a higher weighting to G and C complements, scoring up to a maximum 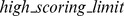. Parameters such as 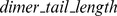, 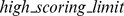 and the weightings of the different bases can be altered to suit the user's needs.

Future plans for the PD5 project include research and development of thermodynamic methods for hairpin and dimerisation detection, the results of which are to be included in a future software release.

### Annealing temperatures

The popular annealing temperature algorithms using thermodynamic data [19], [20] available in Primer3′s *oligoTm* code are included in the PD5 software library, and these have been shown to work well empirically for primers up to 32 bases in length. The annealing temperature class also includes several methods for calculating annealing temperatures using equations devised by Freier [21] and Wallace [22], which can calculate close approximations for longer primers. For the present, the default temperature calculation method used in the primer class is Primer3′s *oligoTm* method.

We aim to increase the range of annealing temperature algorithms available to PD5 in the near future. As an open source project, one of our objectives is to promote the inclusion of good code from other open source projects as reusable modules that increase collective knowledge and understanding of the most important factors that constrain the primer design process. One area of concern is that ideally, primers should have a more or less constant GC content throughout their length, but in instances where this is not possible the presence of many GC residues at the 3′ end can lead to mis-priming. Methods to determine the GC distribution along the length of primers would be beneficial particularly for use with longer primers. We provide an abstract base class for annealing temperature, and 4 derived classes that demonstrate how different temperature calculation algorithms can be incorporated into PD5. The end user can choose their preferred temperature method, but can easily change this for another method later, since all annealing temperature subclasses must implement the same core functionality. New temperature calculation methods can be added as further classes without disrupting existing code.

### Secondary binding and products

PD5 presently has four methods for secondary binding detection. Three methods use third party software: BLAST [23], FASTA [24] and SSEARCH [25]. However, due to their heuristic nature, these methods will occasionally fail to detect significant alignments that can result in mis-priming and the corresponding production of alternative PCR products. This is because these applications are sequence alignment algorithms and work on the probability that a given sequence has significant sequence similarity to a matching sequence on the template. Potential primer binding sites are likely to have extremely varied probabilities of homology and sequence alignment algorithms could overlook primer binding sequences with a high probability of occurrence on the template. This is particularly a problem with small sequence stretches that have evenly spaced mismatches [11]. Furthermore, sequence alignment algorithms are computationally expensive compared with string matching algorithms and are largely unnecessary for the detection of secondary binding. Therefore, we developed a string matching algorithm optimised for the detection of primer binding on DNA sequences, which is included in the *dna_find* class. When tested on the *S. cerevisiae* genome and a plasmid sequence containing the URA3 gene from *Kluyveromyces lactis*, sequence alignment algorithms failed to detect 2% of the primer binding locations on the plasmid sequence, which were correctly detected by the sequence matching algorithm used in the secondary binding detection method in the *dna_find* class.

Secondary primer binding becomes a serious problem if it causes the exponential amplification of an alternative product. PD5 provides a method for PCR product detection. It uses the *dna_find* class to find all binding sites and then checks downstream from the location of each forward binding site for a reverse binding site within a user defined region on the template. The length of the user defined region depends essentially on the PCR extension time (approx. 1000 bases per min). The default length in PD5′s *search_for_pcr_products* method in the *dna_find* class is 3500 bases. This method detects all potential products. One of those products will, of course, be the target region, so it is the occurrence of more than one product that gives an indication of potential secondary product formation. A pseudocode algorithm outlining the basic operation of the product detection method is included in the Information S1(Section S2, Algorithm S1) along with the code and results of a demonstration application to find products in *S. cerevisiae* (Information S1, Section S2, Tables S4 and Figures S3 and S4).

To encourage further secondary binding methods to be added to PD5 we have implemented an abstract base class *nsb*, from which specific implementation classes can be derived, and *dna_find* provides one such example of this.

### Multi-objective optimisation for primers

Multi-objective optimisation problems require an optimal solution to a problem that has characteristics that lie in several independent dimensions. For primer design, we prefer primers that are optimised for some or all of the previously described characteristics, such as suitable sizes and locations, no secondary binding and suitable annealing conditions. As is often the case, a solution that is optimal in all dimensions may not exist and in such cases, a Pareto optimal solution is chosen that takes into account a user specified weighting or priority on the dimensions deemed to be most important.

A multi-objective optimisation algorithm is included as a selectable option in the *sort_candidates* method in the *primer* class. The objective function is given by:
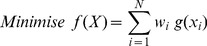
(1)where 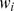 is the user specified weighting and 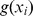 is a non-linear component applied to the value of the characteristic 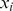. The non-linear component we use is the sigmoid function given by:
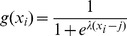
(2)where 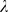 is a gain term used to adjust non-linearity of the sigmoid (default value 1.0), and 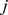 is an offset used to push all characteristic scores into the non-linear region of the sigmoid that provides the best discrimination (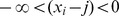). A brief example of non-linear multi-objective optimisation in included in the Information S1 (Section S3).

### Determination of optimum primers

The determination of optimum primers is based on a stable iterative list sorting method where items at the bottom of the list can be rejected. A threshold value can be set so that all items in the list with characteristic values outside of the threshold can be eliminated from the list before re-sorting. This is particularly useful with rule based dimerisation scoring methods, where a score above a defined threshold indicates potential dimerisation. Primers in the sorting list with scores above the threshold are sorted to the bottom of the list and then rejected leaving a subset that contains only items with dimerisation scores below the threshold for subsequent sorting.

We currently use a prioritised sorting method where the priority for sorting according to each characteristic can be varied. This allows the sorting criteria to be changed, and therefore the order of the results, in accordance with the user's requirements. If, for example, the best forward primer we find has more than one binding site on the template, then there is the possibility of secondary product formation if the reverse primer has more than one binding site also. In such a circumstance we can increase the priority of the secondary binding score for the reverse primer so that those potential primers having just one template binding site are placed at the top of the list. In this way primer pairs less likely to make secondary products will be placed at the top of the list. Users who wish to use BLAST, FASTA or SSEARCH for the detection of potential secondary binding sites on the template will find this sorting approach useful to determine primer pairs that potentially form secondary products.

The multi-objective optimisation procedure described above is included in the options for candidate sorting and is best used as the last step in the sorting procedure. This allows for a more precise selection of the remaining candidate primers at the top of the lists previously sorted by the other options.

An application named *basic_app* is included in the PD5 download package that demonstrates the sorting procedure showing a list of primer candidates found, a list of primer candidates after sorting and the final list of candidate primer pairs.

### Comparison with Primer3

Primer3 has far more options than PD5, with over 150 parameters, and a full evaluation/comparison of each of these is beyond the scope of this paper. Primer3 has been extensively used for over 13 years now, and has had many additions during this period. It has a large user base, from occasional non-experts using the web interface, to expert users who need specialist options. In this respect it easily provides a wide range of functionality that PD5 does not provide, including for example, allowing the user to specify that primers must span exon-exon junctions. The two systems share many common features, including methods for calculating annealing temperature, assessing dimer potential, and allowing the user to specify useful constraints such as the required GC composition. They also both provide a web interface, though PD5′s web interface currently has far fewer options. One of the main advantages that PD5 provides but Primer3 does not is an integrated non-specific binding search against a genome sequence. This search (dna_find) does not rely on external services such as BLAST, but is implemented natively, and it is not a heuristic search, so is guaranteed to find all alternative potential binding sites meeting the parameters the user has chosen. The other main focus of PD5 has been on the ability to design and test the properties of long primers, which are increasingly required by the synthetic biology community. This has involved the implementation of dimerisation and annealing temperature methods that are more suited to applications requiring long primers.

## Supporting Information

Information S1 **Further information on the biology methods used for validating primers (including example gels), algorithms and test results from the DNAfind class, and further information on the multi-objective optimisation method used in optimum primer selection.**
(ZIP)
