# Supplementary material for: PD5: A General Purpose Library for Primer Design Software
Source: PLoS One. 2013 Nov 21;8(11):e80156. doi: 10.1371/journal.pone.0080156 (PMC3836914; doi:10.1371/journal.pone.0080156)
Supplement: Information S1 — Further information on the biology methods used for validating primers (including example gels), algorithms and test results from the DNAfind class, and further information on the multi-objective optimisation method used in optimum primer selection. (ZIP) [file pone.0080156.s001.zip]

PD5: a general purpose library for primer design software - supplementary information

Michael C. Riley, Wayne Aubrey, Michael Young and Amanda Clare

S1 Example gels showing PCR results

S1.1 Design of large chimeric primers for coupled PCR

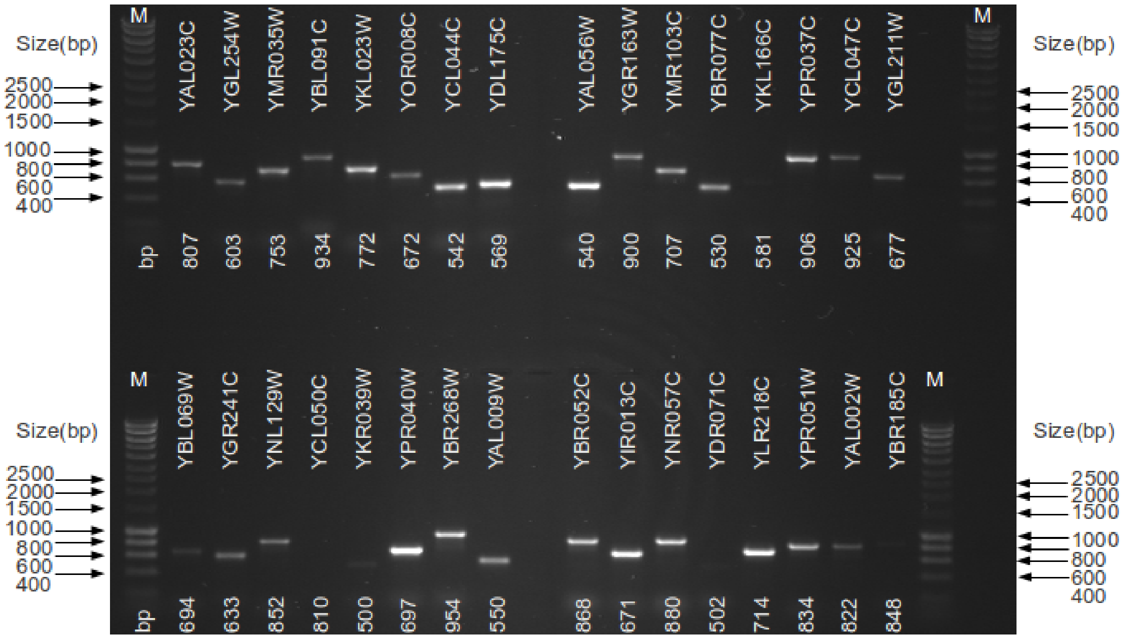

Figure S1: Amplicons obtained from the upstream regions of 32 different *Saccharomyces cerevisiae* ORFs using long PCR primers (equal to or greater than 40 nt) are shown together with their expected sizes (bp).

PCR Reaction Details:

- Quanta Biotech AutoQ thermal cycler
- PCR Tubes: Fisherbrand 0.2 ml flat cap natural. Product No:14230225 PCR Reagents
- GoTaq DNA Polymerase Promega (Cat No. M3171).
- dNTP Mix (10 mM) Promega (Cat No. U1511)

PCR reaction mix:

- GoTaq, 1 μL
- dNTPs (0.2 mM)
- Primers (desalted) were purchased from Invitrogen.
- Forward primer, 2 μL (100 pmol)
- Reverse primer, 2 μL (10 pmol)

- Template Yeast DNA (BY4741 MatA), 1μL (0.05 μg)
- miliQ Water to 50 μL
- Total: 50μL

Yeast DNA purified as described in Qiagen DNeasy Blood and Tissue handbook (cat No. 69504)
PCR reaction mix prepared on ice.
PCR cycle (using Quanta Biotech thermal cycler):

- Heated lid 105 °C
- Initial denaturation 94 °C, 20 sec
- 5 cycles of touchdown PCR where annealing temperature reduced from 65 °C to 60 °C
  - Step 1: 94 °C, 20 sec
  - Step 2: 65-60 °C, 30 sec
  - Step 3: 68 °C, 2 min
- 25 cycles of:
  - Step 1: 94 °C, 20 sec
  - Step 2: 58 °C, 30 sec
  - Step 3: 68 °C, 2 min
- Final extension 72 °C, 5 min
- Hold 10 °C

Electrophoresis Reagents:

- Agarose (Fisher Product No: BP1346-100g)
- SYBR Safe in DMSO (10,000x) Invitrogen (cat. no S33102).
- Tris Base (Sigma Product No:93349)
- Glacial acetic acid (Sigma Product No:537020)
- EDTA (Sigma Product No:431788)

Gel:

- 100 ml 1% agarose supplemented with 4 microL SYBR Safe.
- 10x Tris Acetic Acid EDTA (TAE) buffer (242 g Tris Base, 57.1 ml glacial acetic acid (17.4 M), 100 ml 0.5 M EDTA pH8)
- Electrophoresis Power Pack: Bio-Rad Power Pack basic
- Run Settings 50V for 140 min
- Electrophoresis tank Wide Mini ReadySub-Cell GT Cell Model No. 170-4489EDU
- TAE buffer chilled to 4 °C before use.
- Electrophoresis tank placed on ice for duration of run.
- Gel image captured on Syngene GeneGenius

Primers for S1 can be found in Tables S1 and S2.

S1.2 Design of short primers

PCR Reaction Details:

- Techne TC-512 thermal cycler
- PCR Tubes: Fisherbrand 0.2ml flat cap natural. Product No:14230225

PCR Reagents:

- Eurogentec GoldStar Mix. Product No:PK-0064-02
- DMSO (sigma Product No:472301)

ORF	forward
YAL023C	TTCCTGTATTTGCGTCTGCCACCTACGTGA
YGL254W	AGTGTTGGTGATGAGTTCTCCTCCCTATACCCT
YMR035W	TAGCAGCCTTTACCGCGCAACAAAATCCATATACTATGGACGAG
YBL091C	TGCTTACTTTTCGTTTCCACTTTATCTTGCTGTTCGTTCGAGCAACAGTATTTATT
YKL023W	TTCCCCATATTGGGAACCTGCTCTGCCCTGCTCAGCACGTAG
YOR008C	AAATAATGTGGAGATCTTATCTTGTTTTTCTCTTCTTTATGACA
YCL044C	AAAACGATGTCAGAGGCGTCGTAATTGAAGGTTACCCAACAA
YDL175C	CGTCTACAGAACCCCTGAAGAACACGAAACCTGTAGCCAA
YAL056W	AGGCGCAAATTATTGGGAATAAGACTTGGCTG
YGR163W	AACAGATTCATGAGGAAGAGGAGAGACAACGTCAATT
YMR103C	GATAATGACAAAACAGACACTTTCTGTGGTACTCC
YBR077C	CGTAGGTGTTTGACATTGAATATTTATACCGGCT
YKL166C	CCAGGTACGAGTGATTTAGGAAGGTCTGAAGAAAAGCGACTATGCATT
YPR037C	TGCCCTTATACCGCGTAGTGTAGGTCCAGTCGTACTT
YCL047C	GAGAAATCCTCATTACCCAAGAGCACAAGGAT
YGL211W	AAACTGCTGACGAAGTGGGATGGGATGAGATGATAA
YBL069W	CCTTGTCCCTGTGGAGATAGGTTTCAAATATATCTGGATGACATGTTTGAGGGCGA
YGR241C	TTCTCCAATACTCCAATTGCAACGCACCATATTATCCGGGAGAATACACG
YNL129W	ACTTTCTCCAAAAGGGCCTCAATACATGGTT
YCL050C	CTTTTTTTTATTGTGTTGGTTTTTATATGTTTTGTTATGTATTGTTTATTTTCCCT
YKR039W	TTATTTATTTTTTTTTATACCATTTCCTTTTGATAAGGGGT
YPR040W	GCCGTCCTGCTAGATGTCTGTACGGGATACTTCTCAATCAACTT
YBR268W	CACAATAAGCACTTCAAAGATGTTGAAGAAAACCTGGAACACATGTTTAGGACCC
YAL009W	TTGTCACTACTCAACGTATTCGCACTACTAACACTG
YBR052C	TGTTTGCTTCGTGTTGACCTATTAGCTCACAAGATAGAGCTTGTTTGGAA
YIR013C	CTGTATTAGCTTGTCATCTAAGCCTTCGTCATTGAAGTATCCC
YNR057C	TTGAGATCGGTAACTTCTTCTGCATTCCAATGGTCCTCGAAACCGTT
YDR071C	TTTGTTTTACAACCAAAAGTTTTAAAAAAGTGGATTCATATAAGGTTAG
YLR218C	GTAGTCTTTACGGTAATGGAGTTGGCCCTTG
YPR051W	CAACCAAGGTTCGTCGTCAGTAAGCTAGTTACGGAATGGTAA
YAL002W	AAGCCAGCTGCTAAGTCCATTGTCACTCTA
YBR185C	AATACTTCCTTGCCCCTCTCTCATTAATTCTTGGCCAGTTATAATCGA

Table S1: Forward primers for Figure S1

ORF	reverse
YAL023C	GATTGCTGGACCACGGTTCGAAACAGAATG
YGL254W	TGTTTTGGTGGCCACGTATTCTGGTACCACTTG
YMR035W	TACTGTACAGTACAATGCAACAGCTTCATGAATAGCAGCACGCC
YBL091C	TTTTCAATACGGTAGAGCTTCTACAGTACTTGTTGATGTAAAAGCCCACTTATTAG
YKL023W	GCTTGCACGTTATACCTGGCCGGGATTGGGGCGCTATTCTTG
YOR008C	TATTTAAATAGAATTTTTTATCCTGAGTACTAAATCAGCCAACG
YCL044C	CTTTTATTACGGTAATTGGAAAAGGAGAGGGAATGGAGGAGG
YDL175C	GACTGCGAGGAAGTGATGTAAGCTGCTATCGTTGTACAGG
YAL056W	GCCTATGATTTAGTAATGCGACAATGGACCTG
YGR163W	GTTGTATGTGTATTAGTACCGTTGTCCTGGAGTTTTG
YMR103C	TCTATTTTCTGTAGTTCTTCTTGTCCCCGTTAACC
YBR077C	ATTGCTTCAATATTTTGGATACCGTTTGCTGCTG
YKL166C	TTTGTGCAGGCTCGCTCTTTCCTTGTACAACCACCGATATACAATATG
YPR037C	CCCTTCGTTCCTTGCACAATCTGTCCAGACTTTCCTC
YCL047C	ATTAGTCTGCTAGTGCACGTACTGCTATCAAC
YGL211W	GACGGTTCCTTTGCTTTACTCTTACCATATCGTCTC
YBL069W	TGTTCAACGTTTGCCTCTTGGTATGCAATGTCGTATTTGTAGCTTCCGGAATGCTC
YGR241C	TGTTTCACAAACACGCCTCTATATCTATTATAGGGGTAGCCGAGACAGTC
YNL129W	ATTTTGAGGTTCTACTCTAGCTCACACTTCG
YCL050C	TTTTATTTTAATTTTTACTTTTCTGTTTGTTCTAAAATCTATCTAAACTGGCTTTC
YKR039W	TTTTTATTTCTTTTTTTTGTTTCTTATAAATGTTGCTGTCC
YPR040W	TTTTTCTGGGGAGCTGTTGTGTCTAAAGCTGCCCTTAGGTCATG
YBR268W	CTTCAGTCTTCAGCAGCTATTTCCTTTGCTGTAGATATAACTGCTTTAAGAAGCC
YAL009W	GACCTCCTTTATTTAGCTTTCCACTACCTTTCTTCC
YBR052C	TATCTATGTGGCGTAGTATGTGCTTATAATAGTGAGTAGTATGCTGGAGG
YIR013C	AGCGACCCTGTAATGTTATGTTTCTAGCTAGGAACAGAAAGTG
YNR057C	GACCTGTGCGCTAGTGATTTTTTGAGTTCTTTTCACTGGCCTACTTG
YDR071C	TTTAACTTTTTATTTAATCGTAATGTATTGGTGTTATTTTTTTCTCCGAG
YLR218C	AAATGAAGCGAATGGATGAGAAGAGGGGAGG
YPR051W	GTCCTGACCCAACGCCTTTTCTGTAGTAGTGGCTTTTTTATC
YAL002W	TCTAGGTGTAATGAGTAATGGTCTCGGCAG
YBR185C	CTCATAAGCTTAGCTGAATGGATAGGCTTGCTTTCTGATGGAAATTTG

Table S2: Reverse primers for Figure S1

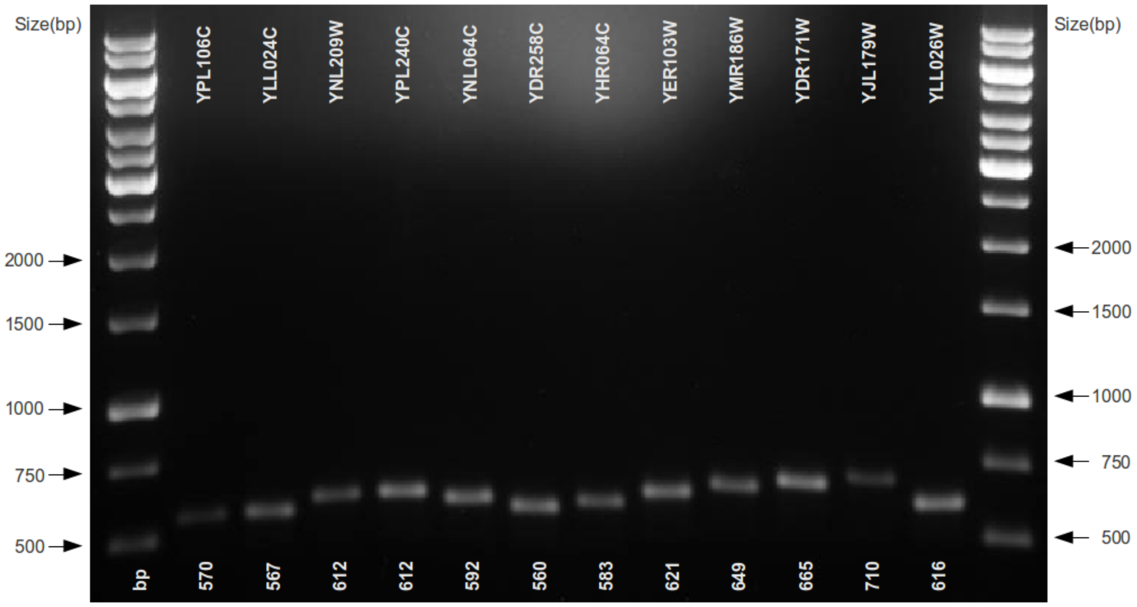

Figure S2: Amplicons confirming the presence of 12 different *Saccharomyces cerevisiae* ORFs using short PCR primers (equal to or greater than 40 nt) are shown together with their expected sizes (bp).

- MiliQ water

PCR Cycle:

- Heater Lid 105C
- Initial denaturation 94 °C, 5 min
- 30 cycles of:
  - Step 1: 92 °C, 10 sec
  - Step 2: 59 °C, 30 sec
  - Step 3: 72 °C, 30 sec
- Final extension 72 °C, 5 min
- Hold 10 °C.

PCR reaction mix:

- GoldStar Mix 25 μL
- Forward Primer (20 pmol)
- Reverse Primer (20 pmol)
- Template Yeast DNA (YSBN 5) 6.44 ng
- DMSO 1.5 μL (3%)
- miliQ water to 50 μL

Yeast DNA purified as described in Qiagen Genomic tip 20G (Product No:10223) handbook. PCR reaction mix prepared on ice.
Electrophoresis Reagents:

- Agarose (Fisher Product No: BP1346-100g)
- Ethidium bromide 10mg/ml (Sigma Product No: E151-10ml)
- Tris Base (Sigma Product No:93349)
- Glacial acetic acid (Sigma Product No:537020)

ORF	forward	reverse
YPL106C	TTTCCAAAGAGCACTACTTGTC	TGTCCTGCTTGACGGTGTC
YLL024C	ATGGGAAGGTGTGGACGAATTG	TTGGCACCCAAGTAAGATTCGG
YNL209W	TTGGGCGACGCAGAGATG	AAGCTGGGACAGTAATGACAG
YPL240C	AATACCAACCAGGTCCTTCCG	GTATTGTTCGTCGTCGTTGCTC
YNL064C	GCCCGAACAAGCGTCTTATTTG	ACCACCACGACCTTCACATTC
YDR258C	AGTTACTGACTCACGCACACAC	AGCTCGACCGATCAAACATGG
YHR064C	TTCTTCGTCGAATGTGATGGTG	AGCTTCCTTTACGGCAGAACC
YER103W	AAGCTCCTTAGTTTGACGACAG	TCGTTGAAATAGGCTGGAACC
YMR186W	TCTCGAACTTCCACCAAGCG	AGAACCACCGGCATTAGATTCC
YDR171W	CTGGAAGGTTCGCGAATTGAG	AACTGTTTGCCTGTTGGTTGG
YJL179W	CCTTAATGATGGCAGCATTCCG	TTTGCAACAGCAGTGCTAACC
YLL026W	GGCGCAAACTTATGCAACC	ACCACGAAGTTCAAGAGCTTG

Table S3: Primers used for Figure S2

- EDTA (sigma Product No:431788)

Gel:

- 100 ml 1% agarose supplemented with 0.04 mg Ethidium bromide.
- 10x Tris Acetic Acid EDTA (TAE) buffer (242 g Tris Base, 57.1 ml glacial acetic acid (17.4 M), 100 ml 0.5 M EDTA pH8)
- Electrophoresis Power Pack: Bio-Rad Power Pack basic
- Run Settings 50V for 290 min
- Electrophoresis tank CBS Scientific Co Model No: MGU-502T
- TAE buffer chilled to 4 °C before use.
- Electrophoresis tank placed on ice for duration of run.
- Gel image captured on UVP Visi-Doc-IT Imaging System.

Primers are shown in Table S3

S2 The DNAfind class

S2.1 Algorithm

An algorithm for the *DNAfind::search_for_pcr_products* is given in Algorithm S1.

S2.2 Examples using the DNAfind class

A brief demonstration using *DNAfind::search_for_pcr_products* was performed on five primer pairs from previous work on the genome of *S. cerevisiae*. A list of these primers is given in Table S4. The code for the small application used to demonstrate the DNAfind class is given in Listing S3 and the results are given in Figure S4. The arrangement of the sequences given in the results for each ORF in Figure S4 is as follows:

```
Start location on sense strand>>>           <<<End location on sense strand
Primer sequence>>>                                     Template sense strand
Complement template antisense strand  <<<Reverse complement primer sequence
```

Note that *DNAfind::search_for_pcr_products* searches for all products including the product we wish to amplify. The default parameters are to inspect 19 base tails, allowing a maximum of 5 mismatches within the 19 bases, to require the first 3 GCs at the 3' end to match, and a

Algorithm S1 DNAfind::search_for_pcr_products

Require: Sequence for each chromosome in FASTA format.
Require: Forward primer 3' tail sequence of length DNAfind::tail_length.
Require: Reverse primer 3' tail sequence of length DNAfind::tail_length.

```
number_of_products = 0
data_count = 0
while chromosome do
  for location = 0 to (chromosome_length − tail_length) do
    // Optimised search of sense strand for primer sequences
    if Sequence match found at location then
      sense_location[data_count] ⇐ Location
      sense_match[data_count] ⇐ Match_sequence
      increment data_count
    end if
  end for
  data_count = 0
  for location = 0 to (chromosome_length − tail_length) do
    // Optimised search of sense strand for the reverse complement of the primer sequences
    if Sequence match found at location then
      antisense_location[data_count] ⇐ Location
      antisense_match[data_count] ⇐ Match_sequence
      increment data_count
    end if
  end for
  for x = 0 to size_of(sense_data_array) do
    for y = 0 to size_of(antisense_data_array) do
      if (antisense_location[y] > sense_location[x]) AND (antisense_location[y] −
      sense_location[x]) < DNAfind :: max_viable_product_length then
        increment number_of_products
        if report_details then
          print Match location details
        end if
      end if
    end for
  end for
end while
return number_of_products
```

ORF	Sequence
YHR163W	TTGTCGCGGACCAGGGGTAAAGCAATTGATGGTGCATTGCCTTC
	CAAGACGGTGCATTTCTGGGCTCCTACTTTGAAATGGGGTCTGG
YPR073C	TTTCGTGTTAACTTTCCCTTCTCAGTTTTCTATCGCTTATCAAAAATCACAGGGTTTC
	TTGTTTCTCTACCAGATTTCTTCACTTTTGACAAACAACCCCTAGAATACAGACTATC
YGR125W	ATTTCTAAATTAAATTTTTGAGATTTTAAACTGTCTTGCTTATAGACC
	AATCTATATAGATATATGTATATAATAAAAGTTTTCAATCTGCTGACC
YJL134W	TTTTTATCTATACAAATGGCTTGTGTCCGCTGGTCTGGTAGG
	CACATTGTCGTCAAAAGGTAATCTCCCCAGAAGTTTTCACTC
YPL262W	GATTGTATCTCTTATCCGTTTAATAGTTAGACTTTATGG
	TTATCCTTCTTATTCTTCTTTGCCATTAATCACTTCTAC

Table S4: Test primer sequences.

maximum product length of 3500 bases.

```
#include <cstdio>
#include <ctime>
#include "../DNAfind.h"
using namespace std;

int main(int argc, char** argv)
{
  int Products = 0;
  clock_t start, stop;

  DNAfind my_dnaf("s_cere_genome.fa");
  my_dnaf.set_max_mismatches(5);
  my_dnaf.set_tail_length(20);
  my_dnaf.set_max_viable_product_length(3500);
  my_dnaf.GC_array_optimisation = TRUE;
  my_dnaf.report_details = TRUE;

  start = clock();
  for(int x = 0; x < 6; x++){
    cout << ORF[x] << ", " << endl;
    Products = my_dnaf.search_for_pcr_products(primer_A[x], primer_B[x]);
    cout << "Products = " << products << endl;
  }
  stop = clock();
  cout << "Processing time: " << stop - start << " microseconds\n";
    return(1);
}
```

Figure S3: Code listing for the small application that uses PD5's *DNAfind::search_for_pcr_products* class for testing six primer pairs from the *S. cerevisiae* genome.

```
YHR163W,
423158>>>                                   <<<423700
ACTTTGAAATGGGGTCTGG>>>           GAAGGCAATGCACCATCAA
ACTTTGAAATGGGGTCTGG           <<<GAAGGCAATGCACCATCAA
Product length 542 on sequence Chr_VIII

490168>>>                                   <<<490624
TTGATGGTGCATTGCCTTC>>>           CCAGACCTGATATTAAAGG
TTCATGTTGCTTGGCCTGC           <<<CCAGACCCCATTTCAAAGT
Product length 456 on sequence Chr_XVI
Products = 2

YPR073C,
215911>>>                                   <<<218934
TCAAAAATCACAGGGTTTC>>>           GCAACCCTGTGATAATTTC
TCCTAAATCACCGGGTTCC           <<<GAAACCCTGTGATTTTTGA
Product length 3023 on sequence Chr_IV

692454>>>                                   <<<693043
TCAAAAATCACAGGGTTTC>>>           GATAGTCTGTATTCTAGGG
TCAAAAATCACAGGGTTTC           <<<GATAGTCTGTATTCTAGGG
Product length 589 on sequence Chr_XVI
Products = 2

YGR125W,
741763>>>                                   <<<742300
AGTTTTCAATCTGCTGACC>>>           GGTCTATAAGCAAGACAGT
AGTTTTCAATCTGCTGACC           <<<GGTCTATAAGCAAGACAGT
Product length 537 on sequence Chr_VII

212225>>>                                   <<<213080
AGTTTTCAATCTGCTGACC>>>           GGACAAAAAGCAAGATACT
AGATCCAAATATGCTGACC           <<<GGTCTATAAGCAAGACAGT
Product length 855 on sequence Chr_XIV
Products = 2

YJL134W,
157639>>>                                   <<<158160
TCCCCAGAAGTTTTCACTC>>>           CCTACCAGACCAGCGGACA
TCCCCAGAAGTTTTCACTC           <<<CCTACCAGACCAGCGGACA
Product length 521 on sequence Chr_X
Products = 1

YPL262W,
46500>>>                                     <<<47315
TGCCATTAATCACTTCTAC>>>           CCATAAAGTCTAACTATTA
TGCCATTAATCACTTCTAC           <<<CCATAAAGTCTAACTATTA
Product length 815 on sequence Chr_XVI
Products = 1

Processing time:  4.66s
```

Figure S4: Console output from a small application that uses PD5's *DNAfind:: search_for_pcr_products* class for testing five example primer pairs for the amplification of upstream regions of ORFs from the *S. cerevisiae* genome. For all five ORFs the PCR products have been detected, but for YHR163W, YPR073C and YGR125W potential secondary products have also been detected.

S3 Non-linear multi-objective optimisation example

The objective function from the main manuscript is given by:

$$Minimise \;\; f(X) = \sum_{i=1}^{N} w_i \, g(x_i) \tag{1}$$

where w_i is the user specified weighting and $g(x_i)$ is a non-linear component applied to the value

of the characteristic x_i. The non-linear component we use is the sigmoid function given by:

$$g(x_i) = \frac{1}{1 + e^{\lambda(x_i - j)}} \tag{2}$$

where λ is a gain term used to adjust non-linearity of the sigmoid (default value 1.0), and j is an offset used to push all characteristic scores into the non-linear region of the sigmoid that provides the best discrimination $(-\infty < (x_i - j) < 0)$.
To demonstrate why the non-linear component of the objective function described in the main manuscript is necessary, consider two hypothetical primers that give the following four dimerisation scores (representing the formation of: hairpins, self dimers, forward primer tail dimerisation with the reverse primer, and reverse primer tail dimerisation with the forward primer, respectively.

Primer A example scores	8	9	9	8
Primer B example scores	8	7	11	8

For this particular example a score of 12 or more indicates a high probability of dimerisation. Using a linear objective function both primers would have an equal score and it would not be possible to discriminate either primer. However, the dimerisation score of 11 in primer B is less desirable since it is closer to the threshold of 12 than the respective score of 9 in primer A. The non-linear component will disproportionately increase higher scores giving primer B a higher score, $f(X)$, than primer A and since we are looking to minimise $f(X)$, primer A will be selected in preference to primer B as required.
